# PREDICTING 10 YEAR MORTALITY WITH MACHINE LEARNING: EVIDENCE FROM NATIONAL SOCIAL LIFE, HEALTH, AND AGING PROJECT

**DOI:** 10.1093/geroni/igad104.1562

**Published:** 2023-12-21

**Authors:** Yiang Li

**Affiliations:** University of Chicago, Chicago, Illinois, United States

## Abstract

The current academic literature extensively used linear logistic models with social, behavioral, and psychological status to predict mortality. However, few address the interdependency of predictors and the imbalance of targets that adversely bias the results. Using the National Social Life Health and Aging Project (NSHAP), we developed two machine learning models predicting the 10-year mortality of older adults in the US. We first used tree-based algorithms of Decision Tree (DT) that account for the interdependency of the social features and decide the splitting nodes and thresholds using entropy gain conditional on the previous splitting predictor to discern disposition status. Second, we used the Fuzzy Support Vector Machine (FSVM) that regards every sample as a node in high-dimensional vector space and splits the nodes with an optimum plane by finding the best linear combination of features to get an optimum prediction accuracy. Additionally, FSVM addresses the target imbalance problem by conducting a more delicate classification of samples with close predicted probabilities of being alive and deceased. Compared to the accuracy rates achieved by the Logistic Regression, our algorithms perform better on the entire population and the population near the class boundary. We also discussed the social and demographic characteristics of the cases whose disposition statuses were either wrongly predicted as deceased or alive by our algorithms. The findings serve important purposes for public health practitioners in accurately understanding the risk and protective factors of mortality in aging.

